# The Primary Duct of *Bothrops jararaca* Glandular Apparatus Secretes Toxins

**DOI:** 10.3390/toxins10030121

**Published:** 2018-03-13

**Authors:** Richard Hemmi Valente, Fernanda Sakai, José Antonio Portes-Junior, Luciana Godoy Viana, Sylvia Mendes Carneiro, Jonas Perales, Norma Yamanouye

**Affiliations:** 1 Laboratório de Toxinologia, Instituto Oswaldo Cruz, FIOCRUZ, Rio de Janeiro RJ 21040-900, Brazil; richardhemmi@gmail.com (R.H.V.), jonasperales@gmail.com (J.P.); 2 Laboratório de Farmacologia, Instituto Butantan, São Paulo SP 05503-900, Brazil; fesakai@gmail.com (F.S.), lucianagodoyviana@gmail.com (L.G.V.); 3 Laboratório de Imunopatologia, Instituto Butantan, São Paulo SP 05503-900, Brazil; portes.junior@butantan.gov.br; 4 Laboratório de Biologia Celular, Instituto Butantan, São Paulo SP 05503-900, Brazil; symcarneiro@gmail.com; 5 Instituto Nacional de Ciência e Tecnologia em Toxinas (INCTTox), CNPq, Brasília DF 71605-170, Brazil

**Keywords:** venom glandular apparatus, primary duct, morphology, secretory cycle, venom production, proteomics, *Bothrops jararaca*

## Abstract

Despite numerous studies concerning morphology and venom production and secretion in the main venom gland (and some data on the accessory gland) of the venom glandular apparatus of Viperidae snakes, the primary duct has been overlooked. We characterized the primary duct of the *Bothrops jararaca* snake by morphological analysis, immunohistochemistry and proteomics. The duct has a pseudostratified epithelium with secretory columnar cells with vesicles of various electrondensities, as well as mitochondria-rich, dark, basal, and horizontal cells. Morphological analysis, at different periods after venom extraction, showed that the primary duct has a long cycle of synthesis and secretion, as do the main venom and accessory glands; however, the duct has a mixed mode venom storage, both in the lumen and in secretory vesicles. Mouse anti-*B. jararaca* venom serum strongly stained the primary duct’s epithelium. Subsequent proteomic analysis revealed the synthesis of venom toxins—mainly C-type lectin/C-type lectin-like proteins. We propose that the primary duct’s toxin synthesis products complement the final venom bolus. Finally, we hypothesize that the primary duct and the accessory gland (components of the venom glandular apparatus) are part of the evolutionary path from a salivary gland towards the main venom gland.

## 1. Introduction

*Bothrops jararaca* (Serpentes, Viperidae) is a Brazilian solenoglyphous venomous snake. The venom glandular apparatus of this snake consists of four distinct parts: the main venom gland, the primary duct, the accessory gland, and the secondary duct that connects to the fang [1].

The main venom gland has a basal-central lumen, which narrows into the primary duct. This duct forms a loop in an orbital area and enters the accessory gland [2]. Near the center of the accessory gland, the primary duct becomes extremely narrow and often displays intricate curvatures before entering the secondary duct [3,4].

Despite the numerous studies concerning the morphology and physiology of secretory cells for venom production and secretion by the main venom gland [5,6,7,8,9,10,11,12,13,14,15] and by the accessory gland [2,4,9,16,17,18,19,20,21], the few studies on the primary duct focused on its morphology [2,3,4,9].

The primary duct epithelium type in rattlesnakes is controversial. In 1966, Kochva and Gans [2] showed that the primary duct of the crotaline snakes is lined by pseudostratified epithelium, whereas 25 years later, Mackessy [9] showed that the primary duct from the *Crotalus viridis oreganus* has a simple epithelium with two cell types—flattened horizontal and columnar secretory.

The aim of this study was to generate a comprehensive morphological (light and electron microscopies), immunohistochemical, and molecular (proteomics) analysis of the primary duct from *Bothrops jararaca*, determining its secretion cycle and assaying for the presence of venom components among the secreted proteins.

Our data showed that the *Bothrops jararaca* primary duct has a long production and secretion cycle (as already established for the main venom and accessory glands) and is able to produce toxins. Moreover, we have determined that the duct’s toxin synthesis pattern is different from those of the main venom and accessory glands. We propose that the primary duct supplies the final venom pool with toxins that are synthesized at low(er) amounts by the other components of the venom glandular apparatus.

## 2. Results and Discussion

### 2.1. Bothrops jararaca Snake Primary Duct’s Morphology

**Figure 1:**
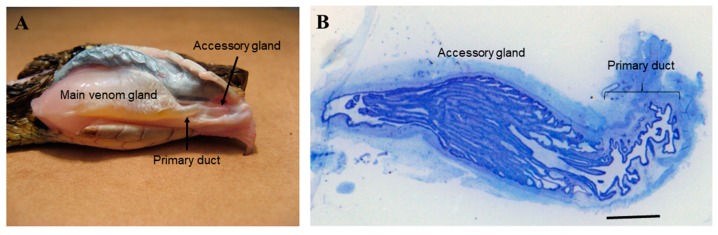
Venom glandular apparatus of the *Bothrops jararaca* snake, showing the primary duct. (**A**) Venom glandular apparatus; (**B**) Histological aspects of the primary duct from 4-day extracted snake stained with toluidine blue. Bar: 1 mm.

The primary duct is located between the main venom gland and the posterior region of the accessory gland (Figure 1, panel A) and it is composed of a folded epithelium (Figure 1, panel B). The main venom and the accessory glands have a long cycle of production and secretion [5,6,7,8,20]. In this study, we analyzed the primary duct at different times after venom extraction. We used the primary duct from snakes that had no venom previously removed (0-day) and from snakes that had their venom manually removed 1 h, and 4, 7 and 15 days before collection.

**Figure 2:**
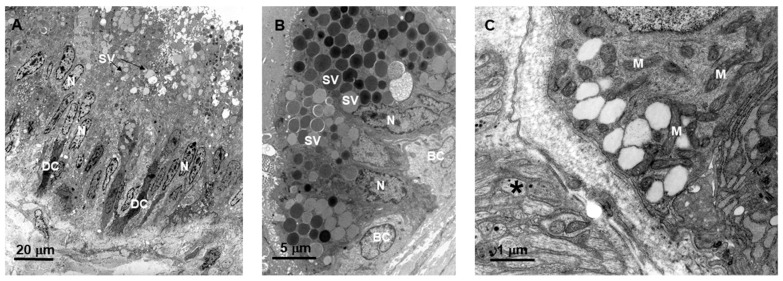
Electron micrograph of the primary duct from non-extracted (0-day) *Bothrops jararaca* snake. BC (basal cell); DC (dark cell); M (mitochondrion); N (nucleus); SV (secretory vesicle); *—nerve terminal. (**A**) Secretory epithelium (pseudostratified) and DC; (**B**) Secretory cells with SV with different electrondensities and BC; (**C**) Mitochondria-rich cells.

Analysis of the primary duct by transmission electron microscopy (TEM) revealed the epithelium to be pseudostratified (Figure 2, panel A), as previously described [2]. The epithelium was mainly formed by secretory cells with microvilli in the apical region. These cells contained secretory vesicles with various electrondensities (Figure 2, panel B). Other cell types observed in the primary duct were basal cells with a round nucleus, mitochondria-rich cells, and dark cells with cytoplasmic projections (Figure 2, panels A to C). Nerve terminals were also observed in the conjunctive space of the basal portion of the primary duct (Figure 2, panel C).

**Figure 3:**
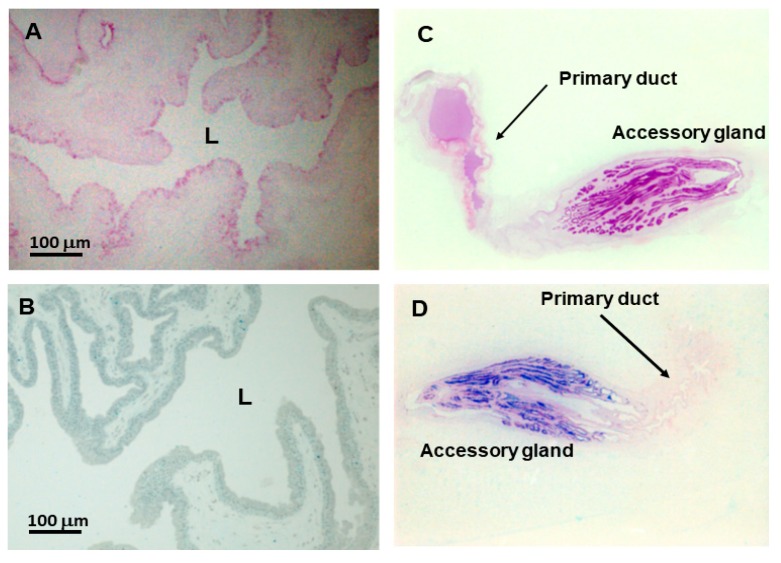
Histological aspects of the primary duct of the *Bothrops jararaca* snake. (**A**,**B**) Primary duct stained with PAS and Alcian blue, respectively; (**C**,**D**) Primary duct and accessory gland from non-extracted (0-day group) snake stained with PAS and Alcian blue, respectively. Regarding the primary duct, notice a weak reactivity to PAS and a negative reactivity to Alcian blue, indicating the presence of neutral polysaccharides only in the secretory epithelium and inside the lumen (L).

Secretory cells from the primary duct had a weak positive reactivity to PAS (Periodic acid-Schiff) staining and a negative reactivity to Alcian blue, indicating the presence of neutral polysaccharides only (Figure 3, panels A and B, respectively). Positive reactivity to PAS was also verified in the primary ducts of *Crotalus atrox*, *C. scutulatus*, and *Trimeresurus erythrurus*. However, the detection of acid polysaccharides seems to be variable, since Alcian blue reacted positively only for *C. atrox* [2]. It should be noted that the lumen of the primary duct from the 0-day group was full of secretion, which was PAS positive (Figure 3, panel C), while in the 4-day group the lumen was empty (data not shown). The storage of venom in *Crotalus oreganus oreganus* also occurs in the primary duct [4]. Therefore, it is not only the main venom gland that stores venom inside the lumen. That is the opposite of what occurs in the accessory gland—the lumen is empty in the 0-day group and full in the 4-day group [20].

**Figure 4:**
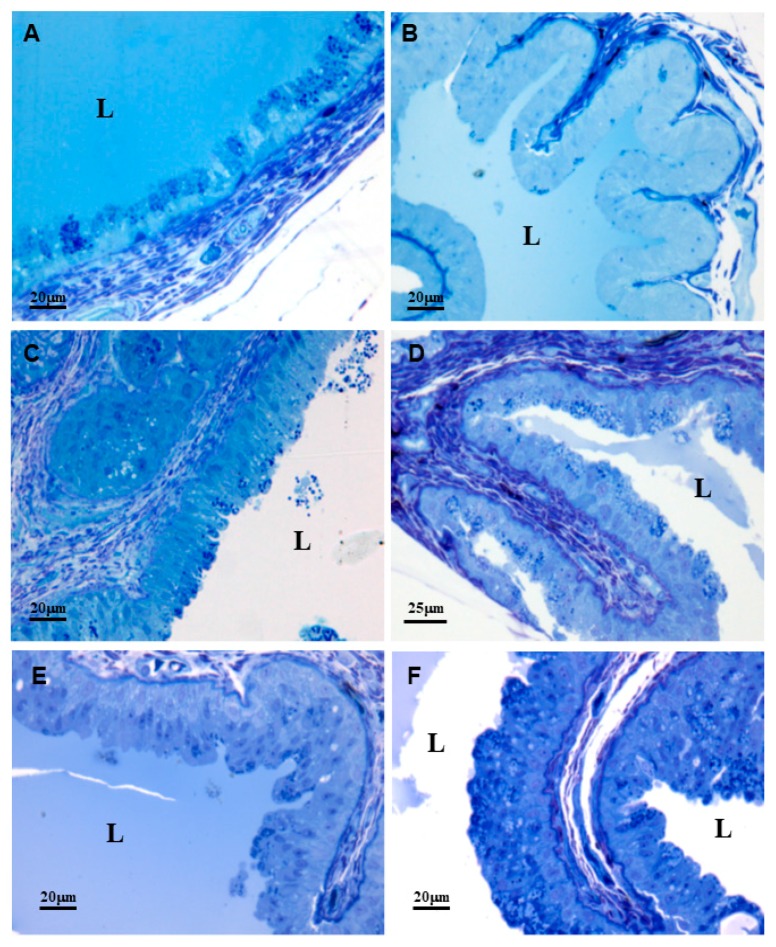
Secretion cycle of the primary duct of the *Bothrops jararaca* snake analyzed with light microscopy. (**A**) Non-extracted (0-day group) snake; (**B**–**D**) One-hour, 4-day, and 7-day extracted snakes; (**E**,**F**) 15-day extracted snake—Lumen (L). Primary duct stained with toluidine blue.

The time course of the synthesis and secretion in the primary duct was analyzed using light microscopy. In the 0-day group, the secretory cells were full of secretory vesicles with different electrondensities and the lumen was full of secretion (Figure 4, panel A). One hour after venom extraction (1-h group), the number of secretory vesicles and the secretion inside the lumen decreased (Figure 4, panel B). In the 4- and 7-day groups, the number of secretory vesicles increased but the lumen was almost empty (Figure 4, panels C and D). In the 15-day group, the number of secretory vesicles and the secretion inside the lumen varied but the morphology was not the same as in the 0-day group (Figure 4, panels E and F), suggesting that the cycle is longer than 15 days. Since the number of secretory vesicles was variable, it seems that during the synthesis and secretion cycle, exocytosis occurs and the secretion is stored in the lumen, as well as in the main venom gland [5,6,7,8,9,10]. After the lumen is completely full, the new synthetized secretory proteins are stored in the secretory vesicles in the cytoplasm, as well as in the accessory gland [20]. We cannot rule out the possibility that the toxins from the lumen of the main venom gland can drain into the primary duct lumen. However, Figure 4 shows that the presence of the secretion inside the lumen is variable for 15 days after venom extraction. If the venom came from the main venom gland, the amount of venom inside the lumen should increase. Furthermore, pressure measurements at different locations along the venom delivery system showed that the venom gland produces suction during relaxation of the extrinsic glandular musculature when the fang is not erected [22]. Therefore, it is unlikely that the venom inside the primary duct lumen comes from main venom gland. Therefore, the primary duct might have two sources of secretion: one stored inside the lumen, which is ejected with the venom inside the lumen of the main venom gland during a bite, and another inside the cells, which needs a stimulus to be exocytosed.

### 2.2. Detection of Venom in the Primary Duct

**Figure 5:**
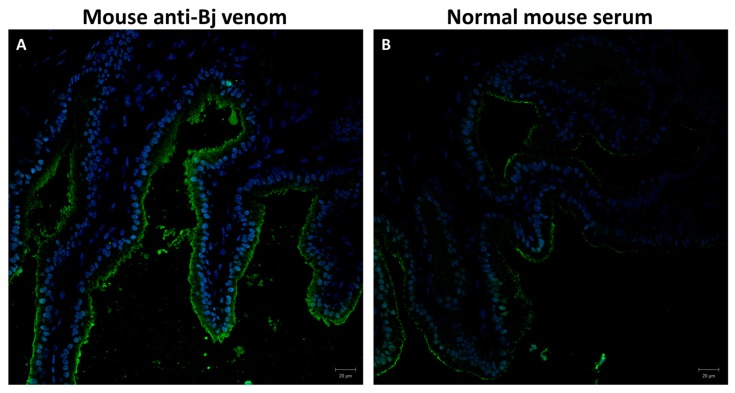
Detection of venom in the secretory epithelium of the primary duct. Sectioned (7 μm thickness) samples were stained with (**A**) mouse anti-*Bothrops jararaca* venom serum or (**B**) normal mouse serum, as a negative control. The nuclear staining (blue) was performed with DAPI (4′,6′-diamidino-2-phenylindole). In panel A, the secretory epithelium was strongly stained (green) at the cytoplasm and the apical region.

To verify whether the primary duct could secrete venom toxin(s)—as happens in the accessory gland [21]—we performed an immunohistochemistry assay. For this assay, we used the primary duct from a snake that had no venom recently extracted (0-day group) to detect the toxins, since we had shown that the amount of secretion inside the vesicles in the cytoplasm was higher than in the other conditions (Figure 4, panel A). Mouse anti-*Bothrops jararaca* venom serum strongly stained the cytoplasm and the apical region of the epithelium of the primary duct (Figure 5, panel A), while normal mouse serum stained very weakly (this was considered the basal level of fluorescence) at the membrane region (Figure 5, panel B). These data demonstrated that the primary duct secretome had at least one of the *B. jararaca* venom components recognized by the antiserum.

### 2.3. Primary Duct’s Secretome Proteomic Analysis

After establishing that the primary duct produced venom or venom fraction(s) (Figure 5, panel A), the next step was to verify whether the toxin families produced by the primary duct were the same as those produced by the main venom and the accessory glands. Based on the morphology study (Figure 4), we decided to use primary ducts from snakes that had no venom previously removed (0-day—secretory cells full of secretory vesicles) and from snakes that had their venom removed manually 4 days before collection (4-day group).

The electrophoretic protein profiles of the primary ducts of *Bothrops jararaca* snake during the secretion production cycle (0-day and 4-day groups) were quite similar according to naked eye inspection (Supplementary Figure S1). For each experimental group, fifteen gel slices were excised from the gels, submitted to *in-gel* digestion with trypsin, and analyzed by nanoESI-LTQ/Orbitrap MS. The quali-quantitave information on the identified proteins, along with their estimated cellular location (www.uniprot.org), is summarized in Supplementary Tables S1A and S2A.

**Figure 6:**
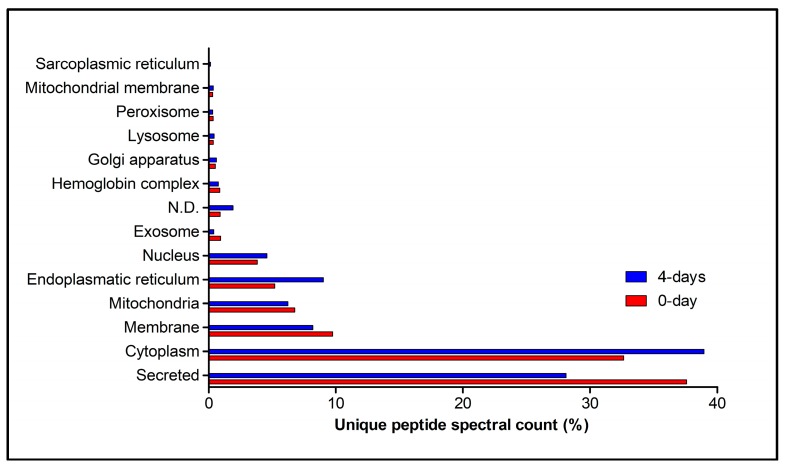
Distribution of proteins identified in the primary duct of the *Bothrops jararaca* snake during the secretory cycle, according to their subcellular location. Represented data are for primary ducts obtained from snakes that had no venom previously removed (0-day group—Red bars) or from snakes that had their venom removed manually 4 days before (4-day group—Blue bars). Classification of protein species were obtained from UniProt database. The distribution of proteins was based on the percentage of unique peptide spectral count for proteins from each subcellular location relatively to the total unique peptide spectral count for all proteins identified in each group (0-day or 4-day). N.D.—not determined.

The proteins identified in the 0-day and 4-day groups were associated with several subcellular locations: cytoplasm, endoplasmic reticulum, exosome, Golgi apparatus, hemoglobin complex, lysosome, membrane, mitochondria, mitochondrial membrane, nucleus, peroxisome, sarcoplasmic reticulum, and secreted proteins. However, the bulk (70.23% and 67.05% for 0 day and 4-day groups, respectively) of the identified proteins were classified as either cytoplasmatic or secreted (Figure 6). In the 0-day group, the majority (37.58%) of proteins detected were secreted, followed by cytoplasmic, membrane, mitochondria, endoplasmic reticulum, and nucleus proteins (32.65%, 9.76%, 6.76%, 5.19%, and 3.82%, respectively). In the 4-day group, the majority of proteins detected were cytoplasmic (38.94%), followed by secreted, endoplasmic reticulum, membrane, mitochondria, and nucleus proteins (28.11%, 9.03%, 8.18%, 6.23%, and 4.58%, respectively). Proteins associated with the other subcellular locations were lower than 1.00% for both groups.

**Figure 7:**
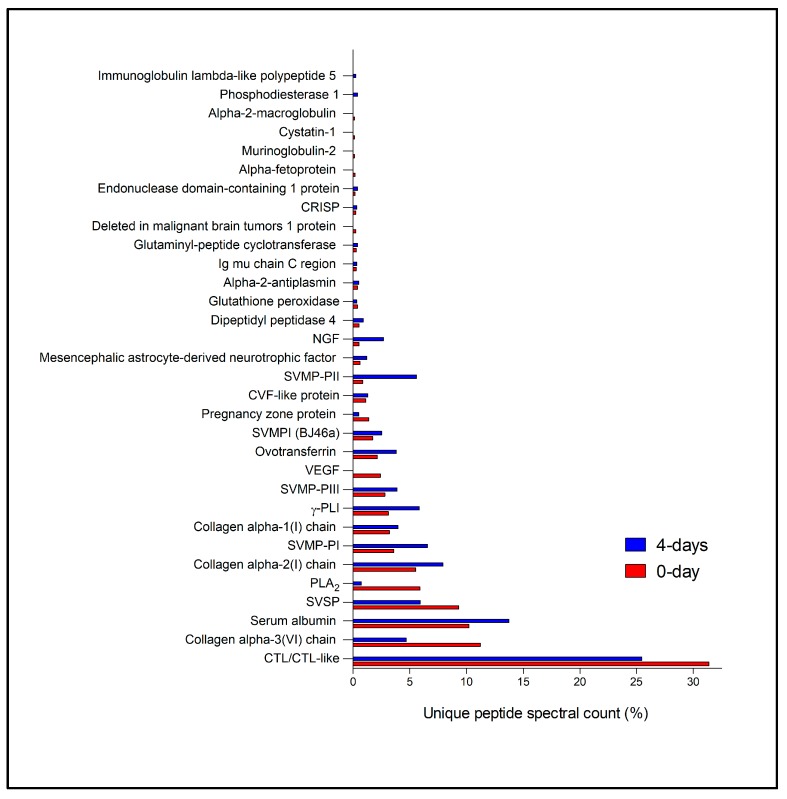
Secreted protein families identified in the primary duct of the *Bothrops jararaca* snake, during its secretory cycle. Red bars—Primary duct from snakes that had no venom previously removed (0-day); Blue bars—Primary duct from snakes that had their venom removed manually 4 days before (4-day). The identified proteins were classified as venom components following the literature [23]. The distribution of proteins was based on the percentage of unique peptide spectral count for proteins from each family relatively to the total unique peptide spectral count for all proteins identified in each group.

Regarding those proteins classified as secreted by the primary duct cells—in either the 0-day or 4-day groups—they encompassed several protein families (Supplementary Tables S1A and S2A), including many known venom components (Supplementary Tables S1B and S2B). For the 0-day group, these venom components were mainly composed of snake CTL/CTL-like (C-type lectin/C-type lectin-like) protein (31.37%), followed by collagen alpha-3(VI) chain (11.22%), serum albumin (10.21%), SVSP (snake venom serine proteinase—9.32%), PLA_2_ (phospholipase A_2_—5.89%), collagen alpha-2(I) chain (5.50%), SVMP-PI (snake venom metalloproteinase, class PI—3.59%), collagen alpha-1(I) chain (3.20%), γ-PLI (gamma type phospholipase A_2_ inhibitor—3.09%), SVMP-PIII (2.81%), VEGF (vascular endothelial growth factor—2.41%), ovotransferrin (2.13%), SVMPI (snake venom metalloproteinase inhibitor—1.74%), pregnancy zone protein (1.40%), and CVF-like protein (Cobra venom factor-like protein—1.12%) (Figure 7). In the 4-day group, the major known venom component detected was also CTL/CTL-like (25.46%), followed by serum albumin (13.74%), collagen alpha-2(I) chain (7.92%), SVPM-PI (6.55%), SVSP (5.90%), γ-PLI (5.82%), SVMP-PII (5.58%), collagen alpha-3(VI) chain (4.69%), collagen alpha-1(I) chain (3.96%), SVMP-PIII (3.88%), ovotransferrin (3.80%), NGF (nerve growth factor—2.67%), SVMPI (2.51%), CVF-like protein (1.29%) and mesencephalic astrocyte-derived neurotrophic factor (1.21%) (Figure 7). Other identified venom components accounted for less than 1.00% in both groups. Due to the limited amount of biological sample, we were not able to perform a robust quantitative experimental approach. Hence, any comparison between 0-day and 4-day group data should be of a qualitative nature. Nonetheless, from a semi-quantitative perspective, and establishing an arbitrary protein abundance fold-change value cut-off of 1.5, in the 0-day group there is a higher abundance of VEGF, PLA_2_, and SVSP, while in the 4-day group SVMP-PII, NGF, γ-PLI, and SVMP-PI are differentially abundant. However, these “trends” should be confirmed by quantitative proteomics experiments before any claims on possible biological roles can be made. In summary, while the expression and synthesis of toxins in the accessory gland is correlated with the main venom gland, being SVMPs the most synthetized toxins [21], in the primary duct the pattern of toxin synthesis is different: synthetized proteins belong predominantly to the CTL/CTL-like family.

**Figure 8:**
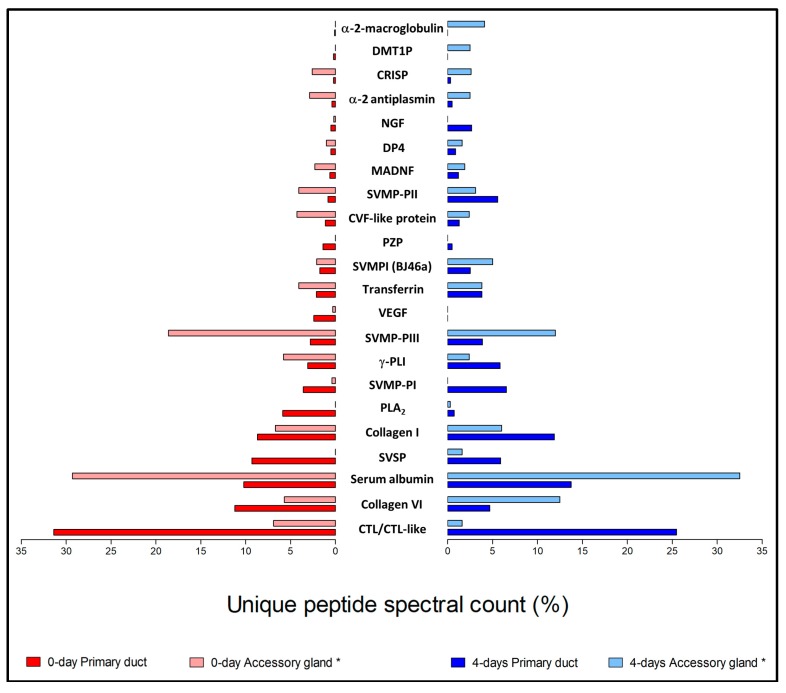
Secreted protein families identified in the primary duct (this work) and in the accessory gland of the *Bothrops jararaca* snake during its secretory cycle. Red bars—Snakes that had no venom previously removed (0-day); Blue bars—Snakes that had their venom removed manually 4 days before (4-day). The identified proteins were classified as venom components following the literature [23]. The distribution of proteins was based on the percentage of unique peptide spectral count for proteins from each family relatively to the total unique peptide spectral count for all proteins identified in each group. DMT1P (deleted in malignant brain tumors 1 protein); DP4 (dipeptidyl peptidase 4); MADNF (mesencephalic astrocyte-derived neurotrophic factor); PZP (pregnancy zone protein). * Data from [21].

When comparing the secreted protein families from the primary duct (this work) and the accessory gland [21], one realizes that among protein families displaying a unique peptide spectral count ≥1%, all are synthetized by both tissues. However, some families are more abundant in the primary duct while others are more abundant in the accessory gland (Figure 8). In the primary duct, for either the 0-day or 4-day groups, CTL/CTL-like, SVSP, PLA_2_, SVMP-PI, and collagen I are more abundant. On the other hand, in the accessory gland, SVMP-PIII, CRISP, CVF-like protein, SVMPI, α-2 antiplasmin, and serum albumin display a higher abundance. Even though one should keep in mind that the proteomic experimental design used in the present paper is far from optimal, the data presented in Figure 8 indicate that not only are the patterns of secreted proteins different, but are also complementary, when the primary duct and the accessory gland are compared.

Taking into consideration our new data, we can now state that three components of the venom glandular apparatus from the *Bothrops jararaca* snake are able to produce toxins—the main venom gland [5,8,24], the accessory gland [21], and the primary duct (this work). Regarding venom storage, the main venom gland synthesizes and stores toxins inside a basal-central lumen [5,8] while, in the accessory gland, all synthesized toxins are stored in secretory vesicles inside the cells [20]. We have now shown that the primary duct displays a mixed mode of storage, that is: it stores toxins inside the lumen (as does the main venom gland) and in secretory vesicles (as does the accessory gland). In other words, the primary duct shares characteristics with both the accessory gland and the main venom gland. It has been reported that the main venom gland evolved from the salivary gland [25]. One could speculate that each part of venom glandular apparatus is the result of an evolutionary process from a salivary gland towards the main venom gland. We have proposed that the accessory gland is an ancillary source of toxins to the snake, since the release of the secretion from the accessory gland starts one hour after venom extraction [20,21], when the production of toxins in the main venom gland is still incipient. Now, we have shown that the secretion of the primary duct contributes to the total venom bolus. As the toxin synthesis pattern is different, a probable function of the primary duct is to provide toxins that are synthesized at lower amounts by the main venom gland and the accessory gland.

## 3. Conclusions

Just as with the main venom and accessory glands, the primary duct displayed a long production and secretion cycle. The secretion produced contained known venom toxins, mainly CTL/CTL-like proteins, and the exocytosis time course observed suggests that these toxins contribute to the total venom bolus, probably complementing the venom with toxins that are synthesized at lower amounts by the main venom gland. Furthermore, the primary duct displays a toxin storage mode that shares characteristics with both the main venom gland and the accessory gland, leading to the hypothesis that each part of the venom glandular apparatus results from the evolutionary process that culminated in the main venom gland.

## 4. Materials and Methods

### 4.1. Animals and Primary Duct

Adult female *Bothrops jararaca* snakes (*n* = 27), weighing 200–400 g, were classified by the Special Laboratory of Zoological Collections at the Instituto Butantan and kept in a room under controlled conditions [26]. Animal care and procedures used were in accordance with the guidelines of the Animal Ethics Committee of the Butantan Institute (374/2007), Biomedical Science Institute of University of São Paulo (138/2009) and the Brazilian Institute for Environment and Renewable Natural Resources, an enforcement agency of the Brazilian Ministry of Environment (IBAMA, License 01/2009). Snakes were left without food for 40 days to prevent loss of venom and to make sure that most of the cells were in the quiescent stage. Fasting periods of 1–2 months are common in snakes living in the wild, but fasts can exceed one year [27]. The snakes were anesthetized with sodium pentobarbital (30 mg/kg, subcutaneous) and decapitated, and the primary ducts were removed and freed from connective tissue [28]. Primary ducts were obtained from snakes that had no venom extracted (0-day, *n* = 12) and from snakes which had their venom manually extracted 1 h (1-h, *n* = 3), 4 (4-day, *n* = 6), 7 (7-day, *n* = 3) or 15 (15-day, *n* = 3) days before they were sacrificed by decapitation. To remove the venom, snakes were anesthetized with sodium pentobarbital (20 mg/kg, subcutaneous), and the venom was removed manually as described in Reference [29].

### 4.2. Light and Electron Microscopy

For light microscopy, the primary ducts were fixed in 4% paraformaldehyde in 0.1 M phosphate buffer (pH 7.2), containing 3.5% sucrose, for at least 8 h at room temperature. The pieces were embedded in Historesin (Leica, Wetzlar, Germany). Sections of 5–6 µm thickness were obtained with a Microm HM 340 E microtome (Microm International GmbH, Walldorf, Germany) and were stained with toluidine blue. Images of the whole gland were taken with an Olympus SZX7 stereomicroscope (Olympus Corporation, Shindiuku, Tokyo, Japan). To determine the presence of acid mucopolysaccharides or neutral polysaccharides, sections were stained with Alcian blue, pH 2.5, and Harry’s hematoxylin or with PAS, respectively. Semithin sections and histological or histochemical sections were analyzed with an Olympus BX51 light microscope coupled to a Q color 5 Olympus digital camera (Olympus Corporation, Shindiuku, Tokyo, Japan).

For electron microscopy, the primary ducts were washed with 0.6% saline solution and 1 mm^3^ fragments were fixed in 2.5% glutaraldehyde and 2% paraformaldehyde in 0.1 M cacodylate buffer (pH 7.2) containing 2% sucrose for at least 2 h at room temperature. Post-fixation was done in 1% osmium tetroxide in 0.1 M cacodylate buffer (pH 7.2) for 1 h at room temperature and the fragments were then placed overnight in 0.5% uranyl acetate containing 13.3% sucrose at 4 °C. Fragments were dehydrated with an ethanol series and propylene oxide and then embedded in Epon resin. Semithin sections (1 µm thickness) were stained with toluidine blue (0.1% toluidine in 1% sodium borate). Ultrathin sections (50–70 nm thickness) were contrasted with uranyl acetate and lead citrate. The grids were examined with an LEO 906 E transmission electron microscope (Zeiss, Oberkochen, Germany) at 80 kV acceleration voltage. Images were acquired by a CCD camera MegaView III (Olympus) through the ITEM—Universal TEM Imaging Platform program (Olympus Soft Imaging Solutions GMBh, Münster, Germany).

### 4.3. Immunohistochemistry Analysis

To detect the presence of toxins, we used the primary duct from snakes that had no venom previously removed (0-day group) since we verified in this study that under this condition the cytoplasm of secretory cells is completely filled with secretory vesicles. The primary duct was included in OCT (optimal cutting temperature) and sections of 7 µm were obtained using cryostat, mounted onto slides, and processed for immunohistochemistry. The slices were fixed in 3.7% formaldehyde for 15 min at room temperature, then incubated with methanol for 5 min at room temperature, washed with PBS containing 0.1% Tween-20 and incubated with blocking solution (1% triton X-100, 5% normal goat serum, 0.5% BSA, 0.5%, glycine 0.5% fish skin gelatin) for 1 h at room temperature. The slices were then incubated with mouse anti-*B. jararaca* venom serum (1:150) for 2 h at room temperature. After washing, the slices were incubated with anti-mouse IgG conjugated with Alexa 488 (*succinimidyl-ester*—Molecular Probes, Eugene, OR, USA) (1:500) and the nucleus labeled with DAPI (Molecular Probes, 1:500). Histochemical sections were analyzed with a confocal microscope (LSM-780-NLO, Zeiss, Germany). For negative control, the slices were incubated with normal mouse serum.

### 4.4. Proteomics

#### 4.4.1. Protein Extraction

Frozen primary ducts (0-day and 4-day; *n* = 3 for each group) were pulverized in liquid nitrogen and homogenized in lysis buffer containing 2 M thiourea, 7 M urea, 4% CHAPS, 30 mM Tris-HCl, pH 8.5, and 1% protease inhibitor cocktail (P8340 Sigma-Aldrich, St Louis, MO, USA). The homogenates were incubated on ice for 1 h and then centrifuged at 12,000× *g* for 10 min at 4 °C. Protein concentration of the supernatants was determined by the Bradford assay [30] using bovine albumin as standard, and the supernatants were kept at −80 °C.

#### 4.4.2. SDS-PAGE (Sodium Dodecyl Sulfate-Polyacrylamide Gel Electrophoresis)

Primary duct protein extracts (30 μg protein/sample) were denatured and reduced in a Laemmli sample buffer for 5 min at 100 °C. Proteins were then subjected to separation on 12% SDS-PAGE with the buffer system described in [31]. After running, the gels were fixed (5% acetic acid, 20% methanol) for at least 30 min and stained with Coomassie Brilliant Blue G (CBB-G). Gels were scanned and the detected band pattern was acquired by Quantity One software (Bio-Rad, Hercules, CA, USA). Technical triplicates of three different extracts per group were analyzed. For one of the replicates, the detected bands were cut and resulted in 15 slices per lane for each group (0-day and 4-day).

#### 4.4.3. In-Gel Trypsin Digestion

Gel slices were processed according to the description in Reference [24] with the modifications described in Reference [21].

#### 4.4.4. Mass Spectrometric Analysis

Samples containing tryptic peptides were resuspended in 10 μL 1% (*v*/*v*) formic acid and submitted to reversed-phase separation on an EASY-nLC II instrument (Thermo Fisher Scientific, Waltham, MA, USA) and mass spectrometric analysis on an LTQ/Orbitrap XL instrument (Thermo Fisher Scientific), as previously described [24].

#### 4.4.5. Data Analysis

MS2 spectra from the 15 samples (originated from 15 SDS-PAGE slices) were pooled, for either 0-day or 4-day groups. Data were analyzed using PEAKS software, version 8.0 build 20160621 (Bioinformatics Solutions Inc., Waterloo, ON, Canada) according to previously described settings [21].

## Supplementary Materials

The following are available online at http://www.mdpi.com/2072-6651/10/3/121/s1, Figure S1: Representative 12% SDS-PAGE of two stages of the secretory cycle from the primary duct of *Bothrops jararaca* snake. Extracts (30 µg) from snakes that had no venom previously removed (0-day) and from snakes that had their venom removed manually 4 days (4-day) were analyzed. Gel was stained with Coomassie brilliant blue G. The lines indicate gel slices submitted to nanoESI-LTQ/Orbitrap XL to identify the proteins. Molecular mass markers (kDa) are indicated on the left, Table S1A: Secreted proteins identified in the extract of the primary duct of *Bothrops jararaca*—No venom previously removed (0-day group), Table S1B: Secreted proteins, identified in the extract of the primary duct from *Bothrops jararaca* that had no venom previously removed (0-day group), which have been described as venom components, Table S2A: Secreted proteins identified in the extract of the primary duct of *Bothrops jararaca*—Venom manually extracted 4 days earlier (4-day group), Table S2B: Secreted proteins, identified in the extract of the primary duct of *Bothrops jararaca* that had venom manually extracted 4 days earlier (4-day group), which have been described as venom components.

## References

[1] Gomes N., Puorto G. (1993). Atlas anatômico de *Bothrops jararaca* Wied, 1824 (Serpentes: Viperidae). Mem. Inst. Butantan.

[2] Kochva E., Gans C. (1966). Histology and histochemistry of venom glands of some Crotaline snakes. Copeia.

[3] Kochva E., Gans C. (1965). The venom gland of *Vipera palestinae* with comments on the glands of some other viperines. Acta Anat..

[4] Mackessy S.P., Baxter L.M. (2006). Bioweapons synthesis and storage: The venom gland of front-fanged snakes. Zool. Anz..

[5] Ben-Shaul Y., Lifshitz S.H., Kochva E., de Vries A., Kochva E. (1971). Ultrastructural aspects of secretion in the venom glands of *Vipera palaestinae*. Toxins of Animal and Plant Origin.

[6] Carneiro S.M., Pinto V.R., Jared C., Lula L.A., Faria F.P., Sesso A. (1991). Morphometric studies on venom secretory cells from *Bothrops jararacussu* (Jararacuçu) before and after venom extraction. Toxicon.

[7] De Lucca F.L., Haddad A., Kochva E., Rothschild A.M., Valeri V. (1974). Protein synthesis and morphological changes in the secretory epithelium of the venom gland of *Crotalus durissus terrificus* at different times after manual extraction of venom. Toxicon.

[8] Kochva E. (1987). The origin of snakes and evolution of the venom apparatus. Toxicon.

[9] Mackessy S.P. (1991). Morphology and ultrastructure of the venom gland of the Northern pacific rattlesnake *Crotalus viridis oreganus*. J. Morphol..

[10] Rotenberg D., Bamberger E.S., Kochva E. (1971). Studies on ribonucleic acid synthesis in the venom glands of *Vipera palaestinae* (Ophidia, Reptilia). Biochem. J..

[11] Luna M.S., Hortencio T.M., Ferreira Z.S., Yamanouye N. (2009). Sympathetic outflow activates the venom gland of the snake *Bothrops jararaca* by regulating the activation of transcription factors and the synthesis of venom gland proteins. J. Exp. Biol..

[12] Kerchove C.M., Carneiro S.M., Markus R.P., Yamanouye N. (2004). Stimulation of the alpha-adrenoceptor triggers the venom production cycle in the venom gland of *Bothrops jararaca*. J. Exp. Biol..

[13] Kerchove C.M., Luna M.S., Zablith M.B., Lazari M.F., Smaili S.S., Yamanouye N. (2008). Alpha1-adrenoceptors trigger the snake venom production cycle in secretory cells by activating phosphatidylinositol 4,5-bisphosphate hydrolysis and ERK signaling pathway. Comp. Biochem. Physiol. A Mol. Integr. Physiol..

[14] Yamanouye N., Britto L.R., Carneiro S.M., Markus R.P. (1997). Control of venom production and secretion by sympathetic outflow in the snake *Bothrops jararaca*. J. Exp. Biol..

[15] Yamanouye N., Carneiro S.M., Scrivano C.N., Markus R.P. (2000). Characterization of beta-adrenoceptors responsible for venom production in the venom gland of the snake *Bothrops jararaca*. Life Sci..

[16] Gans C., Kochva E. (1965). The accessory gland in the venom apparatus of viperid snakes. Toxicon.

[17] Gennaro J.F., Callahan W.P., Lorincz A.E. (1963). The anatomy and biochemistry of a mucus-secreting cell type present in the poison apparatus of the pit viper *Ancistrodon piscivorus piscivorus*. Ann. N. Y. Acad. Sci..

[18] Gennaro J.F., Hall H.P., Casey E.R., Hayes W.K. (2007). Neurotropic effects of venoms and other factors that promote prey acquisition. J. Exp. Zool. A Ecol. Genet. Physiol..

[19] Rhoades R., Lorincz A.E., Gennaro J.F. (1967). Polysaccharide content of the poison apparatus of the cottonmouth moccasin *Agkistrodon piscivorus piscivorus*. Toxicon.

[20] Sakai F., Carneiro S.M., Yamanouye N. (2012). Morphological study of accessory gland of *Bothrops jararaca* and its secretory cycle. Toxicon.

[21] Valente R.H., Luna M.S., de Oliveira U.C., Nishiyama M.Y., Junqueira de Azevedo Ide L., Portes-Junior J.A., Clissa P.B., Viana L.G., Sanches L., Moura da Silva A.M. (2018). *Bothrops jararaca* accessory venom gland is an ancillary source of toxins to the snake. J. Proteom..

[22] Young B.A., Blair M., Zahn K., Marvin J. (2001). Mechanics of venom expulsion in *Crotalus*, with special reference to the role of the fang sheath. Anat. Rec..

[23] Nicolau C.A., Carvalho P.C., Junqueira-de-Azevedo I.L., Teixeira-Ferreira A., Junqueira M., Perales J., Neves-Ferreira A.G., Valente R.H. (2017). An in-depth snake venom proteopeptidome characterization: Benchmarking *Bothrops jararaca*. J. Proteom..

[24] Luna M.S., Valente R.H., Perales J., Vieira M.L., Yamanouye N. (2013). Activation of *Bothrops jararaca* snake venom gland and venom production: A proteomic approach. J. Proteom..

[25] Kardong K. (1982). The evolution of the venom apparatus in snakes from colubrids to viperids & elapids. Mem. Inst. Butantan.

[26] Breno M.C., Yamanouye N., Prezoto B.C., Lazari M.F.M., Toffoletto O., Picarelli Z.P. (1990). Maintenance of the snake *Bothrops jararaca* (Wied, 1824) in captivity. Snake.

[27] Secor S.M., Nagy K. (1994). Bioenergetic correlates of foraging mode fo the snakes *Crotalus cerastes* and *Masticophis flagellum*. Ecology.

[28] Yamanouye N., Kerchove C.M., Moura-da-Silva A.M., Carneiro S.M., Markus R.P. (2006). Long-term primary culture of secretory cells of *Bothrops jararaca* venom gland for venom production in vitro. Nat. Protoc..

[29] Belloumini H.E., Buherl W., Buckley V., Deulofeu V. (1968). Extraction and quantities of venom obtained from some Brazilian snakes. Venomous Animals and Their Venoms.

[30] Bradford M.M. (1976). A rapid and sensitive method for the quantitation of microgram quantities of protein utilizing the principle of protein-dye binding. Anal. Biochem..

[31] Laemmli U.K. (1970). Cleavage of structural proteins during the assembly of the head of bacteriophage T4. Nature.

