# Supplementary material for: The Primary Duct of Bothrops jararaca Glandular Apparatus Secretes Toxins
Source: Toxins (Basel). 2018 Mar 13;10(3):121. doi: 10.3390/toxins10030121 (PMC5869409; doi:10.3390/toxins10030121)

Supplementary Materials: The Primary Duct of *Bothrops jararaca* Glandular Apparatus Secretes Toxins

Richard Hemmi Valente, Fernanda Sakai, José Antonio Portes-Junior, Luciana Godoy Viana, Sylvia Mendes Carneiro, Jonas Perales and Norma Yamanouye


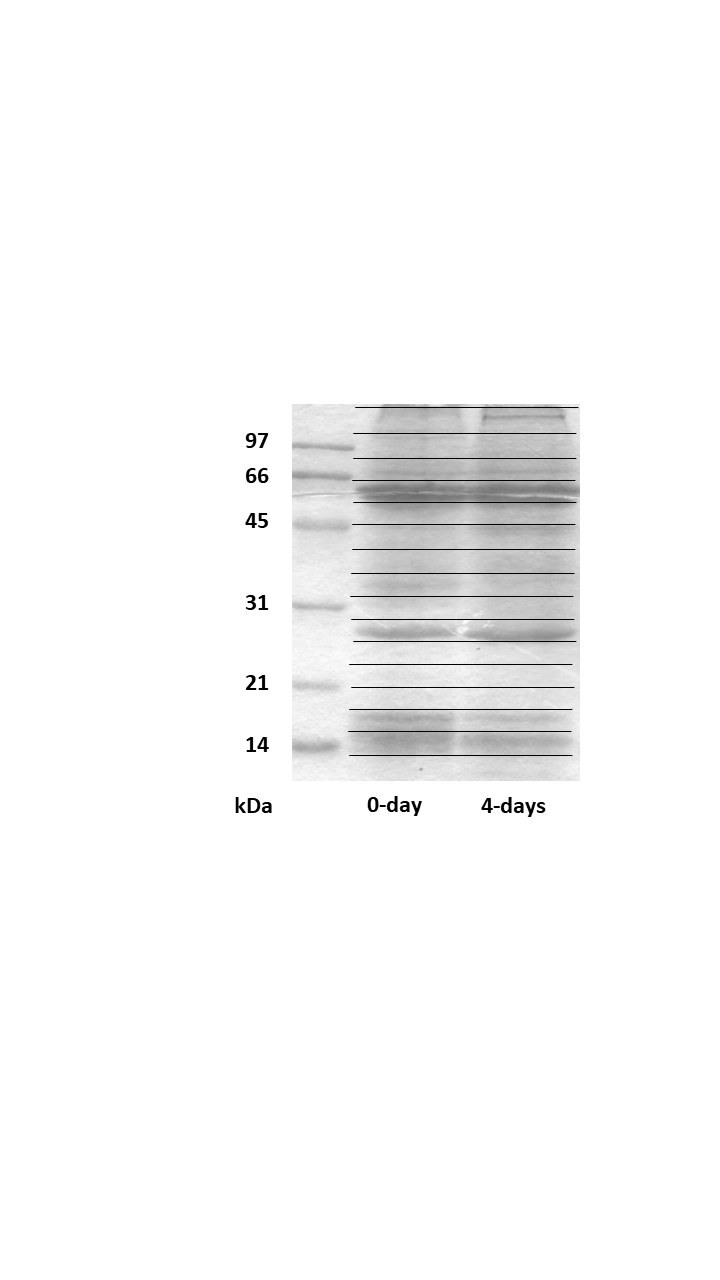

Supplement: Supplementary file 1 [file toxins-10-00121-s001.zip › toxins-279863 Supp proofreading_RHV/toxins-279863 Supp Proofreading_RHV.docx]
